# The effectiveness of vaccine education on vaccine attitudes and knowledge among nursing students: a quasi-experimental study

**DOI:** 10.1590/1980-220X-REEUSP-2025-0027en

**Published:** 2025-07-25

**Authors:** Deniz Sümeyye Yorulmaz Demir

**Affiliations:** 1 Artvin Çoruh University, Faculty of Health Sciences, Nursing Department, Artvin, Turkey.

**Keywords:** Vaccines, Attitude, Knowledge, Students, Nursing, Education, Vacinas, Atitude, Conhecimento, Estudantes de Enfermagem, Educação

## Abstract

**Objective:**

This study aimed to evaluate the effectiveness of vaccine education on vaccine attitudes and knowledge among nursing students.

**Method:**

This research, designed as a quasi-experimental study, was conducted with 68 participants. Vaccine education was delivered in three sessions at one-week intervals. Data were collected using the Personal Characteristics Scale, the Public Attitudes Toward Vaccination Scale Health Belief Model, and the Vaccine Knowledge Test. Data analysis included frequency, percentage, paired t-tests, and effect size (d) to assess differences between means.

**Results:**

Among the participants, 65.2% were female, 14.5% reported difficulties in accessing healthcare services, and only 8.7% followed official websites for vaccine-related information. The study found that vaccine-related knowledge significantly increased after the intervention, with a large effect size (d: 1.755). Vaccine attitudes also showed moderate changes in effect size across various dimensions: perceived susceptibility (d: 0.666), perceived severity (d: 0.682), perceived benefits (d: 0.760), perceived barriers (d: 0.753), and health motivation (d: 0.395). Additionally, the proportion of participants expressing trust in vaccines increased after the educational sessions.

**Conclusion:**

The findings suggest that vaccine education can contribute to improving vaccine attitudes and knowledge among nursing students. Considering the critical role of nursing students as future stakeholders in healthcare delivery, planning and increasing interventions aimed at enhancing their vaccine attitudes and knowledge is essential.

## RESUMO

**Objetivo:**

Este estudo teve como objetivo avaliar a eficácia da educação sobre vacinas nas atitudes e no conhecimento sobre vacinas entre estudantes de enfermagem.

**Método:**

Esta pesquisa, delineada como um estudo quase experimental, foi conduzida com 68 participantes. A educação sobre vacinas foi realizada em três sessões com intervalos de uma semana. Os dados foram coletados por meio da Escala de Características Pessoais, da Escala de Atitudes Públicas em Relação à Vacinação - Modelo de Crenças em Saúde e do Teste de Conhecimento sobre Vacinas. A análise dos dados incluiu frequência, porcentagem, testes t pareados e tamanho do efeito (d) para avaliar as diferenças entre as médias.

**Resultados:**

Entre os participantes, 65,2% eram do sexo feminino, 14,5% relataram dificuldades de acesso aos serviços de saúde e apenas 8,7% acessavam sites oficiais para obter informações relacionadas às vacinas. O estudo constatou que o conhecimento sobre vacinas aumentou significativamente após a intervenção, com um grande tamanho do efeito (d: 1,755). As atitudes em relação à vacina também apresentaram alterações moderadas no tamanho do efeito em várias dimensões: suscetibilidade percebida (d: 0,666), gravidade percebida (d: 0,682), benefícios percebidos (d: 0,760), barreiras percebidas (d: 0,753) e motivação para a saúde (d: 0,395). Além disso, a proporção de participantes que expressaram confiança nas vacinas aumentou após as sessões educativas.

**Conclusão:**

Os resultados sugerem que a educação sobre vacinas pode contribuir para a melhoria das atitudes e do conhecimento sobre vacinas entre estudantes de enfermagem. Considerando o papel crítico dos estudantes de enfermagem como futuros interessados na prestação de cuidados de saúde, é essencial planejar e intensificar intervenções que visem aprimorar suas atitudes e conhecimentos sobre vacinas.

## INTRODUCTION

Vaccines are among the most effective public health interventions proven to protect health, prevent infectious diseases, and control disabilities^(1)^. According to the World Health Organization (WHO), vaccination and immunization programs globally prevent an estimated 2–3 million infant and child deaths annually and significantly reduce disability rates^(2)^. Despite the demonstrated success of vaccines, vaccine hesitancy and refusals have been increasing globally in recent years, leading to a decline in immunization coverage^(3)^. In response to the global decrease in immunization coverage, the WHO has emphasized that vaccine hesitancy and refusals could result in significant public health issues and has established the Strategic Advisory Group of Experts on Immunization (SAGE) to conduct detailed research on this matter. SAGE has identified the primary causes of vaccine hesitancy and refusals as a lack of knowledge about vaccines and immunization and the misinterpretation of available information. The group has stressed that the most critical approach to addressing this issue is education and information dissemination targeted at specific groups^(4)^.

SAGE has identified parents as a key target group for vaccine and immunization education. Additionally, it has been emphasized that healthcare providers and individuals undergoing training in these fields, who will be responsible for future healthcare delivery, are also critical target groups for vaccine and immunization education^(5)^. Ensuring that healthcare providers possess a high level of knowledge and positive attitudes toward vaccines and immunization practices can contribute to the dissemination of accurate and sufficient vaccine information to the public during their professional roles in counseling and education^(6,7)^. Moreover, healthcare providers with high levels of knowledge and positive attitudes toward vaccines and immunization practices can also contribute to increasing immunization rates through appropriate guidance^(8,9)^.

Nurses constitute a significant segment of healthcare providers and are often the first healthcare professionals to interact with the public regarding vaccination and immunization practices, particularly in primary healthcare institutions. Nurses with high levels of knowledge and positive attitudes toward vaccines and immunization can contribute to the dissemination of accurate vaccine information to the community and help increase immunization coverage^(8,10,11)^. In this context, nursing students, who will be responsible for providing nursing services in the future, represent a critical target group for vaccine and immunization education. Evaluating the impact of vaccine and immunization education provided to nursing students is therefore a significant issue.

In Turkey, studies related to vaccination and immunization among nursing students have investigated topics such as immunization status^(12)^, COVID-19 vaccine literacy^(13)^, knowledge levels regarding the Human Papillomavirus (HPV) vaccine^(14)^, and factors influencing vaccination and vaccine knowledge levels^(15)^. However, it has been observed that the impact of vaccine education on nursing students has not been evaluated, which is considered a gap in the literature. Based on this information, the present study was designed to evaluate the effectiveness of vaccine education on vaccine attitudes and knowledge among nursing students in Turkey.

## METHOD

### Purpose and Design of the Study

This study was conducted as a single-group quasi-experimental study to evaluate the effectiveness of vaccine education on vaccine attitudes and knowledge among nursing students. The research hypotheses were defined as follows:

H0_1_: Vaccine education does not affect the vaccine attitudes of nursing students.

H0_2_: Vaccine education does not affect the vaccine knowledge of nursing students.

### Study Group, Inclusion and Exclusion Criteria

The study was conducted at a public university in Turkey between October and November 2024. The participants included second-year nursing students enrolled at the university who volunteered to participate in the study. The decision to include second-year nursing students in the study is based on the fact that these students have already acquired foundational health knowledge, such as anatomy, physiology, and histology, during their first year. This background is essential for the practical experiences they will encounter in hospitals and family health centers in the near future. However, second-year students also require sufficient information for the practices they will perform in family health centers in the coming years. Conversely, first-year nursing students lack sufficient basic health knowledge, and third- and fourth-year nursing students typically learn about vaccines through various courses. Therefore, first-, third-, and fourth-year nursing students were excluded from the study, allowing for a more accurate evaluation of the effects of vaccine education, using only second-year nursing students. Students who did not regularly attend the vaccine education sessions or withdrew their consent were excluded from the study. No sample size calculation was performed. The study group consisted of 68 students who completed the vaccine education program. The students in the study group were in the first semester of the academic year. Education was carried out full-time and face-to-face. There were no courses available on infectious diseases, vaccines, and immunization during the period of the conducted research.

### Intervention and Vaccine Education

The vaccine education sessions were conducted in a classroom at the institution where the study was carried out. The education was delivered as oral presentations in three sessions held one week apart. The sessions took place on Mondays and Fridays from 5:00 PM to 6:00 PM. Students who missed a session were given the opportunity to attend a subsequent session. Repeated sessions have the same content and scope. Each session lasted approximately 60 minutes. Participants’ questions were answered at the end of each session, and each session concluded with the students summarizing the topics discussed. The educational material was shared with the students in the form of PowerPoint presentations.

The content of the vaccine education program was developed through a literature review^(3,6,7,9,11,16,17)^ to enhance students’ vaccine-related knowledge and improve their vaccine attitudes. In addition, opinions were obtained from two experts with doctoral degrees in public health and pediatric nursing in the preparation of the training program. Following the gathering of expert opinions, minimal adjustments were made to the training program. To improve vaccine-related knowledge, the first two sessions of the education covered topics such as the definition of vaccines, vaccine components, the discovery of vaccines, vaccine production processes and safety practices during production, the cold chain, the Vaccine Tracking System, immunity, types of immunity, herd immunity, and adverse effects following vaccination. To enhance vaccine attitudes, the third session included examples of successful vaccination programs (e.g., smallpox vaccine, oral polio vaccine, rabies vaccine, measles vaccine). Additionally, this session addressed the history of vaccine hesitancy, the vaccine-autism phenomenon, the “vaccine Russian roulette” claims, and various initiatives aimed at maintaining immunization rates. The sessions and content of the vaccine education program are detailed in Chart 1. The training program was led by a course instructor at the institution and, at the time of the study, the researcher did not offer a course for second-year nursing students.

**Chart 1:** Content of Vaccine Education Program – Artvin, Turkey, 2024.

Vaccine education content		
Session 1	Session 2	Session 3
Definition of vaccine Discovery and historical development of vaccines Vaccine ingredients Vaccine types Vaccine production processes and phase studies General rules for storing vaccines Cold chain and Vaccine Tracking System	Establishment of vaccination calendars Childhood vaccination calendar and Expanded Immunization Program Immunity, types of immunity (Natural and acquired immunity, active and passive immunity) and community immunity General rules for vaccination Post-vaccination adverse effects and follow-up	Examples of vaccine successes (Smallpox vaccine, oral polio vaccine, rabies vaccine, tuberculosis/TB vaccine, measles vaccine) Vaccine hesitancy and history of vaccine hesitancy Vaccine and Russian roulette, vaccine and autism phenomenon Public health implications and risks of vaccine hesitancy Various practices to prevent vaccine hesitancy and maintain immunization rates

### Data Collection Tools

The data for the study were collected using the Sociodemographic Information Form, the Vaccine Knowledge Test (VKT), and the Public Attitudes Toward Vaccination Scale Health Belief Model.

Sociodemographic Information Form: This form, prepared based on a literature review, includes 17 questions assessing participants’ age, gender, parental education level, education about vaccines, perceptions of vaccination, and vaccinations received during adulthood.

Vaccine Knowledge Test (VKT): The Vaccine Knowledge Test is a self-reported test developed in a doctoral dissertation to assess adults’ knowledge about childhood vaccines. It consists of 28 questions with response options of “True,” “False,” and “I Don’t Know.” Correct answers receive 1 point, while incorrect answers and “I Don’t Know” responses receive 0 points. The total score ranges from 0 to 28, with higher scores indicating greater vaccine-related knowledge. The Cronbach’s alpha for the test was reported as 0.84^(18)^, and in this study, it was calculated as 0.75.

Public Attitudes Toward Vaccination Scale Health Belief Model: This self-reported scale, developed by Koçoğlu-Tanyer et al. (2020) to evaluate public attitudes toward vaccination, consists of 26 items across five subdimensions: perceived susceptibility (5 items), perceived severity (4 items), perceived benefits (5 items), perceived barriers (9 items), and health motivation (5 items). The scale does not yield a total score; instead, each subdimension is evaluated separately. A lower total score in the “Perceived Barriers” subdimension indicates a more positive attitude toward vaccines, while higher scores in the other subdimensions reflect more positive attitudes. The Cronbach’s alpha values for the subdimensions were reported as 0.90, 0.89, 0.86, 0.87, and 0.85, respectively^(19)^. This study calculated the Cronbach’s alpha values as 0.80, 0.83, 0.85, 0.76, and 0.83, respectively.

### Data Collection Process

The data for the study were collected between November 1 and 30, 2024. Initial measurements were taken and pre-test data were collected after obtaining consent from the students in the study group. Following the completion of the vaccine education program, second measurements were taken and post-test data were collected. The average time between pre-test and post-test measurements is 4–5 weeks. The average time the tools required to complete data collection was 5–7 minutes.

### Data Analysis

The research data were analyzed using the Statistical Package for Social Sciences (SPSS) v.29.0. The descriptive characteristics and immunization status of the students were presented via frequencies and percentages. To assess whether the data met the assumptions of normal distribution, skewness and kurtosis values were examined for each variable, and the histograms were interpreted. The analysis confirmed that the dataset met the assumptions of normal distribution, and parametric tests were used. The difference between pre-test and post-test results was analyzed using a paired t-test. To evaluate the effect of the vaccine education, effect size was calculated based on the “difference between two means (d)” and interpreted as follows: 0.01–0.20: small effect, 0.20–0.50: medium effect, 0.80 and above: large effect^(20)^. During the research process, data from one participant who completed the pre-test but did not complete the vaccine education were excluded from the dataset during the analysis. There were no missing data in the study, and no data imputation methods were applied. A significance level of 0.05 was employed to interpret all analysis results.

### Ethical Considerations

Ethical approval (Number: E-18457941-050.99-152637, Date: 14.10.2024) and institutional permission (Number: E-82587833-605-154420, Date: 28.10.2024) were obtained prior to the study. Before the research began, nursing students were informed about the study, and consent was obtained from those who volunteered to participate. The training program was conducted entirely on a voluntary basis and was not part of any formal course. No grades were assigned to the participating students, and participation in the program was entirely optional. Additionally, the names of participants and their results were maintained in anonymity. This study was conducted in accordance with the principles of the Declaration of Helsinki.

## RESULTS

Among the participants, 65.2% were female, 60.9% had mothers who were primary school graduates, 31.9% lacked social security, and 14.5% experienced difficulties in accessing healthcare services. Additionally, 76.8% had not received prior vaccine education, and only 8.7% followed official websites for vaccine-related information. Moreover, 17.4% had a healthcare worker in their family and 34.8% had a child aged 0-5 in their family (Table 1).

**Table 1:** Descriptive characteristics of participants – Artvin, Turkey, 2024 (n = 69).

Characteristics	n	%
Age: 20.84 ± 1.38 (Min: 19, Max: 26)		
**Gender**		
Female	45	65.2
Male	24	34.8
**Mother’s education level**		
Primary school	42	60.9
Middle school	18	26.1
High school	7	10.1
University or higher	2	2.9
**Father’s education level**		
Primary school	25	36.2
Middle school	14	20.3
High school	20	29.0
University or higher	10	14.5
**Social security**		
Yes	47	68.1
No	22	31.9
**Difficulty in Accessing Healthcare Services**		
Yes	10	14.5
No	59	85.5
**Family Member Working in Healthcare**		
Yes	12	17.4
No	57	82.6
**Presence of a Child Aged 0-5 in the Family**		
Yes	24	34.8
No	45	65.2
**Receiving Vaccine Education**		
Yes	16	23.2
No	53	76.8
**Following Official Websites for Vaccine-Related Information**		
Yes	6	8.7
No	63	91.3

n: number, %: percentage

An examination of certain vaccination characteristics revealed that 75.4% of participants had received the tetanus vaccine, 42% had received the Hepatitis A and B vaccines, %17.4 had received the Influenza vaccine, and only 1.4% had received the HPV vaccine (Table 2).

**Table 2:** Vaccine characteristics of participants – Artvin, Turkey, 2024 (n = 69).

Vaccines	n	%
**Tetanus vaccine**		
Administered	52	75.4
Not Administered	17	24.6
**Hepatitis A vaccine**		
Administered	29	42.0
Not Administered	40	58.2
**Hepatitis B vaccine**		
Administered	29	42.0
Not Administered	40	58.2
**Influenza vaccine**		
Administered	12	17.4
Not Administered	57	82.6
**HPV vaccine**		
Administered	1	1.4
Not Administered	68	98.6

n: number, %: percentage

The evaluation of the pre- and post-education vaccination attitudes of the participants revealed a significant increase in each sub-dimension of the “Public Attitude Towards Vaccination Scale-Health Belief Model” (Perceived Susceptibility pre-test: 16.24 ± 2.45, post-test: 17.76 ± 2.10; Perceived Severity pre-test: 15.69 ± 2.69, post-test: 17.42 ± 2.37; Perceived Benefits pre-test: 19.88 ± 3.16, post-test: 22.14 ± 2.77; Perceived Barriers pre-test: 21.31 ± 4.89, post-test: 17.18 ± 6.01; Health Motivation pre-test: 19.36 ± 3.15, post-test: 20.65 ± 3.37). The effect of vaccination education on vaccination attitudes was found to be moderate (Perceived Susceptibility d: 0.666, Perceived Severity d: 0.682, Perceived Benefits d: 0.760, Perceived Barriers d: 0.753, Health Motivation d: 0.395). When participants’ pre- and post-education vaccination knowledge was assessed, a significant increase was observed in the total score of the Vaccination Knowledge Test (pre-test: 13.79 ± 4.55, post-test: 21.69 ± 4.45), and the effect of vaccination education on vaccination knowledge was found to be large (d: 1.755) (Table 3).

**Table 3:** Pre- and Post-Education vaccination attitude and knowledge scores of participants – Artvin, Turkey, 2024.

Variables	Pre-education Mean ± SD	Post-education Mean±SD	Test value p	%95 CI	d
**Vaccination Attitudes**					
Perceived Susceptibility	16.24 ± 2.45	17.76 ± 2.10	t: –4.918 p = <0.001	–2.139 – –0.904	0.666
Perceived Severity	15.69 ± 2.69	17.42 ± 2.37	t: –5.104 p = <0.001	2.398 – –1.050	0.682
Perceived Benefits	19.88 ± 3.16	22.14 ± 2.77	t: –5.397 p = <0.001	–3.096 – –1.424	0.760
Perceived Barriers	21.31 ± 4.89	17.18 ± 6.01	t: 6.255 p = <0.001	2.812 – 5.448	0.753
Health motivation	19.36 ± 3.15	20.65 ± 3.37	t: –3.163 p = 0.002	–2.103 – –0.476	0.395
**Vaccine Knowledge**	13.79 ± 4.55	21.69 ± 4.45	t: –12.681 p < 0.001	–9.141 – –6.655	1.755

Mean: Arithmetic Mean; SD: Standard Deviation; t: Paired Samples t-test; p: Significance Level; 95% CI: Confidence Interval; d: Effect Size.

In the study, in addition to vaccination attitudes and knowledge, the thoughts and trust of nursing students regarding vaccines were also examined. The findings revealed an increase in the proportion of participants who trusted vaccines (pre-education: 29%, post-education: 58%) and who believed vaccines were beneficial and necessary (pre-education: 33.3%, post-education: 69.5%). Conversely, the proportion of participants who considered vaccines to be partially safe (pre-education: 63.9%, post-education: 42.0%) and those who did not trust vaccines (pre-education: 7.2%, post-education: 0%) decreased (Table 4).

**Table 4:** Pre- and Post-Education trust in and thoughts about vaccines – Artvin, Turkey, 2024 (n = 69).

Variables	Pre-education		Post-education	
	n%		n%	
**Thoughts on vaccines**				
Beneficial and necessary	23	33.3	47	69.5
Beneficial but not all necessary	43	62.3	21	30.4
Harmful and unnecessary	–	–	–	–
No opinion	3	4.3	–	–
**Confidence in vaccines**				
Yes	20	29.0	40	58.0
Partially	44	63.8	28	42.0
No	5	7.2	–	–

n: number, %: percentage

## DISCUSSION

Although vaccines are proven to be effective in preventing infectious diseases and controlling disabilities, vaccine hesitancy has globally increased in recent years, leading to a decline in immunization rates^(21)^. SAGE emphasizes the importance of providing information to individuals involved in providing healthcare to prevent vaccine hesitancy and highlights the necessity of such informational efforts targeting these groups^(4)^. In this context, nursing students represent a critical target group for education on vaccines and immunization, making it essential to evaluate the impact of educational interventions designed for that demographic^(3)^. While various studies have assessed the impact of vaccine education on nursing students in different countries^(11,22)^, the absence of such research in Turkey constitutes a gap in the literature. Therefore, this study aims to evaluate the impact of vaccine education on nursing students in Turkey in terms of their attitudes toward vaccines and their vaccine-related knowledge.

In the study, the impact of vaccine education on vaccine attitudes was evaluated initially, and the results indicated an increase in the positive attitudes of nursing students toward vaccines following the training. The vaccine education program included topics such as the discoveries of Edward Jenner and John Salk, the reduction in infectious disease cases and mortality rates following vaccine development, visuals of children disabled due to infectious diseases, the benefits of vaccines, the threats posed by declining immunization rates to child and public health, and a recent rabies case in the country where failure to report a dog bite and receive vaccination resulted in death. These topics included in the vaccine education program are thought to have improved nursing students’ attitudes toward vaccines. Similarly, the literature contains findings that align with our results, reporting that vaccine education programs designed for students are effective in fostering positive attitudes regarding vaccines^(7,22,23)^.

Various models that aim to explain attitude-behavior processes suggest that attitudes are one of the primary determinants of behavior, and fostering positive attitudes toward a subject can increase the likelihood of appropriate behaviors^(24)^. Attitudes toward vaccines can influence not only an individual’s own vaccination behavior, but also the attitudes and behaviors of others regarding vaccination in turn^(6)^. Nurses with highly positive attitudes toward vaccines may be more willing to counsel parents and communities about vaccines and encourage vaccination. By fostering positive attitudes toward vaccines among nurses, more individuals within the community could be provided with vaccine-related counseling, leading to significant gains in promoting vaccination and increasing immunization rates^(25)^. In this context, planning initiatives to improve students’ attitudes toward vaccines, raising awareness about the severity of vaccine-preventable diseases, and providing information regarding the benefits of vaccines are crucial.

Another evaluation criterion in the study was the impact of vaccine education on vaccine-related knowledge. The study findings revealed that students’ vaccine-related knowledge demonstrated a significant increase with a high effect size following the education program. During the vaccine educational sessions, particular emphasis was placed on topics such as vaccine components, mechanisms of action, diseases prevented by vaccines, general rules for vaccine storage, preparation of childhood vaccination schedules, and adverse effects following immunization. It is believed that participants’ detailed knowledge in these areas contributed to overall improvement in vaccine-related knowledge. Similar to our findings, previous studies have reported that vaccine education programs designed for students effectively enhance vaccine-related knowledge^(11,26,27,28)^. Having sufficient knowledge about vaccines plays a critical role in providing accurate information during education and counseling sessions and in encouraging correct vaccine decisions. Ensuring nurses possess adequate vaccine-related knowledge is essential for enabling the public, particularly parents, to access accurate information about vaccines, addressing vaccine hesitancy, and improving immunization rates^(29)^. Additionally, given that nurses are often the first healthcare professionals to engage with the community in primary care services^(10)^, it is crucial to plan initiatives aimed at improving nursing students’ vaccine-related knowledge and to incorporate vaccine education into nursing curricula. Furthermore, in line with the recommendations of the SAGE^(17)^, vaccine education programs should also address the escalating issue of vaccine hesitancy and the public health threats it poses^(17)^.

In addition to vaccine attitudes and knowledge, the study also examined nursing students’ perceptions of vaccines and their confidence in vaccines. The findings revealed a significant increase in the proportion of participants who trusted vaccines and believed that vaccines are a beneficial practice following the study’s educational intervention. The vaccine education program covered topics such as vaccine production processes, phase studies, post-administration monitoring, the cold chain, the Vaccine Tracking System implemented in Turkey, and the benefits achieved through vaccination. It is believed that including these topics in the program positively influenced nursing students’ perceptions of vaccines and their confidence in them. Furthermore, the increase in nursing students’ vaccine-related knowledge and the improvement in their vaccine attitudes during the education program may have contributed to the enhancement of their perceptions and confidence in vaccines. This finding is particularly significant as it suggests that vaccine education can reduce vaccine hesitancy and strengthen confidence in vaccines. Similar results have been reported in the literature, where vaccine education programs were found to reduce vaccine hesitancy and increase vaccine confidence among students^(23,26)^. Additionally, studies have demonstrated that educational interventions targeting vaccines other than childhood immunizations, such as the Human Papilloma Virus (HPV) and COVID-19 vaccines, improve students’ confidence in vaccines and reduce vaccine hesitancy^(30,31)^. Considering that nursing students will be future healthcare professionals and that highly knowledgeable nurses can contribute to increasing immunization rates, planning initiatives to enhance their vaccine confidence is essential. Moreover, future vaccine education planning should focus on raising awareness of the benefits of vaccines and the achievements gained through vaccination, as this plays a critical role in fostering positive perceptions of vaccines and maintaining trust in them. This intervention may be important in encouraging parents and the community to vaccinate students who will be responsible for providing nursing services in the future by enhancing their awareness of the importance of vaccines.

## LIMITATIONS

Although the results of the study contribute to the literature, they also exhibit certain limitations. First, the study was conducted with a group of students who volunteered to take part in the vaccine education program, and no randomization was performed. Second, there was no control group in the study, preventing a comparison of results obtained from the experimental group with a control group. Additionally, vaccine confidence was assessed as a categorical variable. It was not evaluated using a validated and reliable measurement tool. Implementing the education program in a traditional format complicates the generalization of the results. Moreover, this intervention was implemented as a one-time application and its long-term effects were not examined. Despite these limitations, the findings are significant as they demonstrate that vaccine education can enhance vaccine-related knowledge, improve attitudes toward vaccines, and contribute to increasing vaccine confidence.

## CONCLUSION

In this study, conducted to evaluate the impact of vaccine education on nursing students, it was concluded that vaccine-related knowledge significantly increased with a high effect size, and attitudes toward vaccines improved with a medium effect size after the intervention. Moreover, vaccine hesitancy decreased, and there was a notable increase in the proportion of participants who believed vaccines were beneficial and necessary. Based on these results, the study’s hypotheses (H0_1_: Vaccine education has no effect on the vaccine attitudes of nursing students. H0_2_: Vaccine education has no effect on the vaccine-related knowledge of nursing students) were rejected.

Considering that nursing students are future healthcare professionalsand that nurses are often the primary source of information for the public on vaccines and other health topics, it is recommended to plan educational initiatives to enhance their knowledge of vaccines. It is also recommended to incorporate vaccine and immunization topics into nursing curricula. Furthermore, addressing the current state of immunization and the potential public health threats posed by increasing vaccine hesitancy would be crucial. Future research may investigate vaccine hesitancy among nursing students and assess the impact of vaccine education in randomized controlled trials. Additionally, the long-term effects of vaccine education on vaccine-related knowledge and vaccine attitudes may be assessed.

## Data Availability

Research data are available upon reasonable request from the corresponding author.
