# Supplementary material for: The effectiveness of vaccine education on vaccine attitudes and knowledge among nursing students: a quasi-experimental study
Source: Rev Esc Enferm USP. 2025 Jul 25;59:e20250027. doi: 10.1590/1980-220X-REEUSP-2025-0027en (PMC12306190; doi:10.1590/1980-220X-REEUSP-2025-0027en)
Supplement: Translation

# Translation: Eficacia de la educación sobre vacunas en las actitudes y los conocimientos sobre vacunas de los estudiantes de enfermería: un estudio cuasi experimental

**DOI:** 10.1590/1980-220X-REEUSP-2025-0027es

**Authors:** Deniz Sümeyye Yorulmaz Demir

**Affiliations:** 1 Artvin Çoruh University, Faculty of Health Sciences, Nursing Department, Artvin, Turkey.

**Keywords:** Vacunas, Actitud, Conocimiento, Estudiantes de Enfermería, Educación

## Abstract

**Objetivo:**

Este estudio tuvo como objetivo evaluar la efectividad de la educación sobre vacunas en las actitudes y el conocimiento sobre vacunas entre estudiantes de enfermería.

**Método:**

Esta investigación, diseñada como un estudio cuasi-experimental, se realizó con 68 participantes. La educación sobre vacunas se impartió en tres sesiones con intervalos de una semana. Los datos se recopilaron mediante la Escala de Características Personales, la Escala de Actitudes Públicas hacia la Vacunación Modelo de Creencias de Salud y la Prueba de Conocimiento sobre Vacunas. El análisis de datos incluyó frecuencia, porcentaje, pruebas t pareadas y tamaño del efecto (d) para evaluar las diferencias entre medias.

**Resultados:**

Entre los participantes, el 65,2% eran mujeres, el 14,5% reportó dificultades para acceder a los servicios de salud y solo el 8,7% consultaba sitios web oficiales para obtener información sobre vacunas. El estudio encontró que el conocimiento sobre vacunas aumentó significativamente después de la intervención, con un tamaño del efecto elevado (d: 1,755). Las actitudes hacia las vacunas también mostraron cambios moderados en la magnitud del efecto en diversas dimensiones: susceptibilidad percibida (d: 0,666), gravedad percibida (d: 0,682), beneficios percibidos (d: 0,760), barreras percibidas (d: 0,753) y motivación para la salud (d: 0,395). Además, la proporción de participantes que expresaron confianza en las vacunas aumentó después de las sesiones educativas.

**Conclusión:**

Los hallazgos sugieren que la educación sobre vacunas puede contribuir a mejorar las actitudes y los conocimientos sobre vacunas entre los estudiantes de enfermería. Considerando el papel crucial de los estudiantes de enfermería como futuros actores clave en la prestación de servicios de salud, es esencial planificar e incrementar las intervenciones dirigidas a mejorar sus actitudes y conocimientos sobre vacunas.

## INTRODUCCIÓN

Las vacunas se encuentran entre las intervenciones de salud pública más eficaces que se ha demostrado que protegen la salud, previenen las enfermedades infecciosas y controlan las discapacidades^(1)^. Según la Organización Mundial de la Salud (OMS), se calcula que los programas de vacunación e inmunización evitan en todo el mundo entre 2 y 3 millones de muertes de lactantes y niños al año y reducen significativamente las tasas de discapacidad^(2)^. A pesar del éxito demostrado de las vacunas, en los últimos años han aumentado en todo el mundo las dudas y las negativas a vacunarse, lo que ha provocado un descenso de la cobertura de inmunización^(3)^. En respuesta a la disminución mundial de la cobertura de inmunización, la OMS ha hecho hincapié en que la indecisión y el rechazo a las vacunas podrían dar lugar a importantes problemas de salud pública y ha creado el Grupo Estratégico Consultivo de Expertos en Inmunización (SAGE en la sigla en inglés) para llevar a cabo investigaciones detalladas sobre esta cuestión. El SAGE ha identificado como causas principales de la indecisión y el rechazo a la vacunación la falta de conocimientos sobre las vacunas y la inmunización y la interpretación errónea de la información disponible. El grupo ha subrayado que el enfoque más crítico para abordar esta cuestión es la educación y la difusión de información dirigida a grupos específicos^(4)^.

El SAGE ha identificado a los padres como un grupo objetivo clave para la educación sobre vacunas e inmunización. Además, se ha hecho hincapié en que los profesionales sanitarios y las personas en formación en estos campos, que serán responsables de la futura prestación de asistencia sanitaria, también son grupos objetivo fundamentales para la educación sobre vacunas e inmunización^(5)^. Garantizar que los profesionales sanitarios posean un alto nivel de conocimientos y actitudes positivas hacia las vacunas y las prácticas de inmunización puede contribuir a la difusión de información precisa y suficiente sobre vacunas al público durante sus funciones profesionales de asesoramiento y educación^(6,7)^. Además, los profesionales sanitarios con altos niveles de conocimiento y actitudes positivas hacia las vacunas y las prácticas de inmunización también pueden contribuir a aumentar las tasas de inmunización mediante una orientación adecuada^(8,9)^.

Las enfermeras y enfermeros constituyen un segmento importante del personal de salud y a menudo son los primeros profesionales que interactúan con el público en relación con las prácticas de vacunación e inmunización, especialmente en los centros de atención primaria. Las enfermeras con altos niveles de conocimiento y actitudes positivas hacia las vacunas y la inmunización pueden contribuir a la difusión de información precisa sobre vacunas a la comunidad y ayudar a aumentar la cobertura de inmunización^(8,10,11)^. En este contexto, los estudiantes de enfermería, que serán los responsables de prestar los servicios de enfermería en el futuro, representan un grupo objetivo fundamental para la educación sobre vacunas e inmunización. Por lo tanto, evaluar el impacto de la educación sobre vacunas e inmunización impartida a los estudiantes de enfermería es una cuestión importante.

En Turquía, los estudios relacionados con la vacunación y la inmunización entre los estudiantes de enfermería han investigado temas como el estado de inmunización^(12)^, los conocimientos sobre la vacuna COVID-19^(13)^, los niveles de conocimientos sobre la vacuna contra el virus del papiloma humano (VPH)^(14)^ y los factores que influyen en los niveles de vacunación y de conocimientos sobre la vacuna^(15)^. Sin embargo, se ha observado que no se ha evaluado el impacto de la educación sobre vacunas en los estudiantes de enfermería, lo que se considera una laguna en la bibliografía. Basándose en esta información, el presente estudio se diseñó para evaluar la eficacia de la educación sobre vacunas en las actitudes y los conocimientos sobre vacunas entre los estudiantes de enfermería de Turquía.

## MÉTODO

### Propósito y Diseño del Estudio

Este estudio se llevó a cabo como un estudio cuasiexperimental de un solo grupo para evaluar la eficacia de la educación sobre vacunas en las actitudes y los conocimientos sobre vacunas entre los estudiantes de enfermería. Las hipótesis de la investigación se definieron como sigue

H0_1_: La educación sobre vacunas no afecta a las actitudes hacia las vacunas de los estudiantes de enfermería.

H0_2_: La educación sobre vacunas no afecta a los conocimientos sobre vacunas de los estudiantes de enfermería.

### Grupo de Estudio, Criterios de Inclusión y Exclusión

El estudio se realizó en una universidad pública de Turquía entre octubre y noviembre de 2024. Los participantes fueron estudiantes de segundo año de enfermería matriculados en la universidad que se ofrecieron voluntariamente a participar en el estudio. La decisión de incluir a estudiantes de segundo año de enfermería en el estudio se basa en el hecho de que estos estudiantes ya han adquirido conocimientos básicos de salud, como anatomía, fisiología e histología, durante su primer año. Esta base es esencial para las experiencias prácticas que encontrarán en hospitales y centros de salud familiar en un futuro próximo. Sin embargo, los estudiantes de segundo año también necesitan información suficiente para las prácticas que realizarán en los centros de salud familiar en los próximos años. Por el contrario, los estudiantes de enfermería de primer año carecen de suficientes conocimientos básicos sobre salud, y los estudiantes de enfermería de tercer y cuarto año suelen aprender sobre vacunas a través de diversos cursos. Por lo tanto, se excluyó del estudio a los estudiantes de enfermería de primer, tercer y cuarto año, lo que permitió una evaluación más precisa de los efectos de la educación sobre vacunas, utilizando únicamente a estudiantes de enfermería de segundo año. Los estudiantes que no asistieron regularmente a las sesiones de educación sobre vacunas o retiraron su consentimiento fueron excluidos del estudio. No se calculó el tamaño de la muestra. El grupo de estudio estaba formado por 68 estudiantes que completaron el programa de educación sobre vacunas. Los estudiantes del grupo de estudio cursaban el primer semestre del año académico. La educación se llevó a cabo a tiempo completo y de forma presencial. No había cursos disponibles sobre enfermedades infecciosas, vacunas e inmunización durante el periodo de la investigación realizada.

### Intervención y Educación Sobre Vacunas

Las sesiones de educación sobre vacunas se llevaron a cabo en un aula de la institución donde se realizó el estudio. La educación se impartió en forma de presentaciones orales en tres sesiones celebradas con una semana de intervalo. Las sesiones tuvieron lugar los lunes y viernes de 17:00 a 18:00 horas. Los alumnos que faltaron a una sesión tuvieron la oportunidad de asistir a una sesión posterior. Las sesiones repetidas tienen el mismo contenido y alcance. Cada sesión duraba aproximadamente 60 minutos. Al final de cada sesión se respondía a las preguntas de los participantes y los estudiantes resumían los temas tratados. El material educativo se compartió con los alumnos en forma de presentaciones de PowerPoint.

El contenido del programa de educación sobre vacunas se desarrolló mediante una revisión bibliográfica^(3,6,7,9,11,16,17)^ para aumentar los conocimientos de los estudiantes sobre vacunas y mejorar sus actitudes hacia las mismas. Además, se obtuvieron opiniones de dos expertos con doctorados en salud pública y enfermería pediátrica en la preparación del programa de formación. Tras recabar las opiniones de los expertos, se introdujeron ajustes mínimos en el programa de formación. Para mejorar los conocimientos relacionados con las vacunas, las dos primeras sesiones de la formación trataron temas como la definición de las vacunas, los componentes de las vacunas, el descubrimiento de las vacunas, los procesos de producción de las vacunas y las prácticas de seguridad durante la producción, la cadena de frío, el Sistema de Seguimiento de Vacunas, la inmunidad, los tipos de inmunidad, la inmunidad de rebaño y los efectos adversos tras la vacunación. Para mejorar la actitud ante las vacunas, la tercera sesión incluyó ejemplos de programas de vacunación que han tenido éxito (por ejemplo, la vacuna contra la viruela, la vacuna oral contra la poliomielitis, la vacuna contra la rabia y la vacuna contra el sarampión). Además, en esta sesión se abordaron la historia de la indecisión ante las vacunas, el fenómeno vacuna-autismo, las afirmaciones sobre la “ruleta rusa de las vacunas” y diversas iniciativas destinadas a mantener las tasas de inmunización. Las sesiones y el contenido del programa de formación sobre vacunas se detallan en la Cuadro 1. El programa de formación fue dirigido por un instructor del curso en la institución y, en el momento del estudio, el investigador no ofrecía un curso para estudiantes de segundo año de enfermería.

**Cuadro 1:** Contenido del programa de educación sobre vacunas – Artvin, Turquía, 2024.

Contenido del programa de educación sobre vacunas		
Sesión 1	Sesión 2	Sesión 3
Definición de vacuna Descubrimiento y desarrollo histórico de las vacunas Ingredientes de las vacunas Tipos de vacunas Procesos de producción de vacunas y estudios de fase Normas generales para el almacenamiento de vacunas Cadena de frío y sistema de seguimiento de vacunas	Establecimiento de calendarios de vacunación Calendario de vacunación infantil y Programa Ampliado de Inmunizaciones Inmunidad, tipos de inmunidad (inmunidad natural y adquirida, inmunidad activa y pasiva) e inmunidad comunitaria Normas generales de vacunación Efectos adversos posvacunales y seguimiento	Ejemplos de éxito de las vacunas (vacuna contra la viruela, vacuna oral contra la polio, vacuna contra la rabia, vacuna contra la tuberculosis/TB, vacuna contra el sarampión) Reticencia a las vacunas e historial de la misma Vacuna y ruleta rusa, vacuna y fenómeno del autism Implicaciones y riesgos para la salud pública de la reticencia a las vacunas Diversas prácticas para prevenir la reticencia a las vacunas y mantener las tasas de inmunización

### Instrumentos de Recolección de Datos

Los datos para el estudio se recogieron utilizando el Formulario de Información Sociodemográfica, el Test de Conocimientos sobre Vacunas (Vaccine Knowledge Test VKT) y la Escala de Actitudes del Público hacia la Vacunación Modelo de Creencias sobre la Salud.

Formulario de información sociodemográfica: Este formulario, elaborado a partir de una revisión bibliográfica, incluye 17 preguntas que evalúan la edad, el sexo, el nivel de educación de los padres, la educación sobre vacunas, las percepciones sobre la vacunación y las vacunas recibidas durante la edad adulta de los participantes.

Prueba de conocimientos sobre vacunas (VKT): El Test de Conocimientos sobre Vacunas es una prueba de autoinforme desarrollada en una tesis doctoral para evaluar los conocimientos de los adultos sobre las vacunas infantiles. Consta de 28 preguntas con opciones de respuesta “Verdadero”, “Falso” y “No lo sé”. Las respuestas correctas reciben 1 punto, mientras que las respuestas incorrectas y “No sé” reciben 0 puntos. La puntuación total oscila entre 0 y 28, y las puntuaciones más altas indican un mayor conocimiento relacionado con las vacunas. El alfa de Cronbach para la prueba se registró como 0,84^(18)^, y en este estudio se calculó como 0,75.

Escala de actitudes públicas hacia la vacunación Modelo de creencias sobre la salud (Public Attitudes Toward Vaccination Scale Health Belief Model): Esta escala autoinformada, desarrollada por Koçoğlu-Tanyer et al. (2020) para evaluar las actitudes del público hacia la vacunación, consta de 26 ítems a través de cinco subdimensiones: susceptibilidad percibida (5 ítems), gravedad percibida (4 ítems), beneficios percibidos (5 ítems), barreras percibidas (9 ítems) y motivación para la salud (5 ítems). La escala no ofrece una puntuación total en, sino que cada subdimensión se evalúa por separado. Una puntuación total más baja en la subdimensión “Barreras percibidas” indica una actitud más positiva hacia las vacunas, mientras que puntuaciones más altas en las otras subdimensiones reflejan actitudes más positivas. Los valores alfa de Cronbach para las subdimensiones fueron de 0,90, 0,89, 0,86, 0,87 y 0,85, respectivamente^(19)^. Este estudio calculó los valores alfa de Cronbach en 0,80, 0,83, 0,85, 0,76 y 0,83, respectivamente.

### Proceso de Recolección de Datos

Los datos para el estudio se recogieron entre el 1 y el 30 de noviembre de 2024. Se tomaron las medidas iniciales y se recogieron los datos previos a la prueba tras obtener el consentimiento de los alumnos del grupo de estudio. Tras la finalización del programa de educación sobre vacunas, se tomaron las segundas mediciones y se recopilaron los datos posteriores a la prueba. El tiempo medio entre las mediciones pre-test y post-test es de 4–5 semanas. El tiempo medio que necesitaron las herramientas para completar la recogida de datos fue de 5–7 minutos.

### Análisis de Los Datos

Los datos de la investigación se analizaron utilizando el Paquete Estadístico para las Ciencias Sociales (SPSS) v.29.0. Las características descriptivas y el estado de inmunización de los alumnos se presentaron mediante frecuencias y porcentajes. Para evaluar si los datos cumplían los supuestos de una distribución normal, se examinaron los valores de asimetría y curtosis de cada variable y se interpretaron los histogramas. El análisis confirmó que el conjunto de datos cumplía los supuestos de distribución normal y se utilizaron pruebas paramétricas. La diferencia entre los resultados anteriores y posteriores a la prueba se analizó mediante una prueba t pareada. Para evaluar el efecto de la educación sobre la vacuna, se calculó el tamaño del efecto basándose en la “diferencia entre dos medias (d)” y se interpretó de la siguiente manera: 0,01–0,20: efecto pequeño, 0,20–0,50: efecto medio, 0,80 y más: efecto grande^(20)^. Durante el proceso de investigación, se excluyeron del conjunto de datos durante el análisis los datos de un participante que realizó la prueba previa pero no completó la educación sobre la vacuna. No faltaron datos en el estudio y no se aplicaron métodos de imputación de datos. Se empleó un nivel de significación de 0,05 para interpretar todos los resultados de los análisis.

### Consideraciones Éticas

Antes del estudio se obtuvieron la aprobación ética (Número: E-18457941-050.99-152637, Fecha: 14.10.2024) y el permiso institucional (Número: E-82587833-605-154420, Fecha: 28.10.2024). Antes de comenzar la investigación, se informó a los estudiantes de enfermería sobre el estudio y se obtuvo el consentimiento de aquellos que se ofrecieron a participar. El programa de formación se llevó a cabo de forma totalmente voluntaria y no formaba parte de ningún curso formal. No se asignaron calificaciones a los estudiantes participantes, y la participación en el programa fue totalmente opcional. Además, los nombres de los participantes y sus resultados se mantuvieron en el anonimato. Este estudio se realizó de acuerdo con los principios de la Declaración de Helsinki.

## RESULTADOS

Entre los participantes, el 65,2% eran mujeres, el 60,9% tenían madres con estudios primarios, el 31,9% carecían de seguridad social y el 14,5% tenían dificultades para acceder a los servicios sanitarios. Además, el 76,8% no había recibido formación previa sobre vacunas, y sólo el 8,7% seguía las páginas web oficiales para obtener información relacionada con las vacunas. Por otra parte, el 17,4% tenía un trabajador sanitario en su familia y el 34,8% tenía un niño de 0 a 5 años en su familia (Tabla 1).

**Tabla 1:** Características descriptivas de los participantes – Artvin, Turquía, 2024 (n = 69).

Características	n	%
Edad: 20,84 ± 1,38 (Mín: 19, Máx: 26)		
**Sexo**		
Mujer	45	65.2
Masculino	24	34.8
**Nivel de estudios de la madre**		
Estudios primarios	42	60.9
Enseñanza media	18	26.1
Enseñanza secundaria	7	10.1
Universidad o superior	2	2.9
**Nivel de estudios del padre**		
Estudios primarios	25	36.2
Enseñanza media	14	20.3
Enseñanza secundaria	20	29.0
Universidad o superior	10	14.5
**Seguridad social**		
Sí	47	68.1
No	22	31.9
**Dificultad para acceder a los servicios sanitarios**		
Sí	10	14.5
No	59	85.5
**Miembro de la familia que trabaja en la sanidad**		
Sí	12	17.4
No	57	82.6
**Presencia de un niño de 0-5 años en la familia**		
Sí	24	34.8
No	45	65.2
**Educación sobre vacunas**		
Sí	16	23.2
No	53	76.8
**Sigue los sitios web oficiales para obtener información relacionada con las vacunas**		
Sí	6	8.7
No	63	91.3

n: número, %: porcentaje

El examen de determinadas características de la vacunación reveló que el 75,4% de los participantes habían recibido la vacuna antitetánica, el 42% habían recibido las vacunas contra la Hepatitis A y B, el %17,4 habían recibido la vacuna contra la Gripe y sólo el 1,4% habían recibido la vacuna contra el VPH (Tabla 2).

**Tabla 2:** Características de las vacunas de los participantes – Artvin, Turquía, 2024 (n = 69).

Vacunas	n	%
**Vacuna antitetánica**		
Administrada	52	75.4
No administrada	17	24.6
**Vacuna contra la hepatitis A**		
Administrada	29	42.0
No administrada	40	58.2
**Vacuna contra la hepatitis B**		
Administrada	29	42.0
No administrada	40	58.2
**Vacuna antigripal**		
Administrada	12	17.4
No administrada	57	82.6
**Vacuna VPH**		
Administrada	1	1.4
No administrada	68	98.6

n: número, %: porcentaje

La evaluación de las actitudes de vacunación de los participantes antes y después de la formación reveló un aumento significativo en cada subdimensión de la “Escala de actitud pública hacia la vacunación-Modelo de creencias sobre la salud” (Susceptibilidad percibida pre-test: 16,24 ± 2,45; posprueba: 17,76 ± 2,10; Severidad percibida pre-test: 15,69 ± 2,69, postest: 17,42 ± 2,37; Beneficios percibidos pretest: 19,88 ± 3,16, post-test: 22,14 ± 2,77; Barreras Percibidas pre-test: 21,31 ± 4,89, post-test: 17,18 ± 6,01; Motivación para la salud pre-test: 19,36 ± 3,15, postest: 20,65 ± 3,37). El efecto de la educación sobre vacunación en las actitudes hacia la vacunación resultó ser moderado (Susceptibilidad percibida d: 0,666, Gravedad percibida d: 0,682, Beneficios percibidos d: 0,760, Barreras percibidas d: 0,753, Motivación para la salud d: 0,395). Cuando se evaluaron los conocimientos sobre vacunación de los participantes antes y después de la educación, se observó un aumento significativo en la puntuación total de la Prueba de Conocimientos sobre Vacunación (antes de la prueba: 13,79 ± 4,55, después de la prueba: 21,69 ± 4,45), y se comprobó que el efecto de la educación sobre vacunación en los conocimientos sobre vacunación era grande (d: 1,755) (Tabla 3).

**Table:** Tabla 3 – Puntuaciones de actitud y conocimientos sobre vacunación de los participantes antes y después de la educación – Artvin, Turquía, 2024.

Variables	Pre-educación media ± DE	Post-educación media ± DE	Valor de la prueba p	%95 IC	d
**Actitudes ante la vacunación**					
Susceptibilidad percibida	16.24 ± 2.45	17.76 ± 2.10	t: –4,918 p = <0,001	–2.139 – –0.904	0.666
Gravedad percibida	15.69 ± 2.69	17.42 ± 2.37	t: –5,104 p = <0,001	–2.398 – –1.050	0.682
Beneficios percibidos	19.88 ± 3.16	22.14 ± 2.77	t: –5,397 p = <0,001	–3.096 – –1.424	0.760
Barreras percibidas	21.31 ± 4.89	17.18 ± 6.01	t: 6.255 p = <0.001	2.812 – 5.448	0.753
Salud motivación	19.36 ± 3.15	20.65 ± 3.37	t: –3,163 p = 0,002	–2.103 – –0.476	0.395
**Conocimientos sobre vacunas**	13.79 ± 4.55	21.69 ± 4.45	t: –12,681 p < 0,001	–9.141 – –6.655	1.755

Media: Media aritmética; DE: desviación estándar; t: Prueba t de muestras pareadas; p: Nivel de significación; IC 95%: Intervalo de confianza; d: Tamaño del efecto.

En el estudio, además de las actitudes y los conocimientos sobre vacunación, también se examinaron los pensamientos y la confianza de los estudiantes de enfermería con respecto a las vacunas. Los resultados revelaron un aumento de la proporción de participantes que confiaban en las vacunas (preeducación: 29%, poseducación: 58%) y que creían que las vacunas eran beneficiosas y necesarias (preeducación: 33,3%, poseducación: 69,5%). Por el contrario, disminuyó la proporción de participantes que consideraban que las vacunas eran parcialmente seguras (preeducación: 63,9%, poseducación: 42,0%) y los que no confiaban en las vacunas (preeducación: 7,2%, poseducación: 0%) (Tabla 4).

**Tabla 4:** Confianza antes y después de la educación y pensamientos sobre las vacunas – Artvin, Turquía, 2024 (n = 69).

Variables	Pre-educación		Post-educación	
	n	%	n	%
**Opiniones sobre las vacunas**				
Beneficiosas y necesarias	23	33.3	47	69.5
Beneficiosas pero no todas necesarias	43	62.3	21	30.4
Perjudiciales e innecesarias	–	–	–	–
Sin opinión	3	4.3	–	–
**Confianza en las vacunas**				
Sí	20	29.0	40	58.0
Parcialmente	44	63.8	28	42.0
No	5	7.2	–	–

n: número, %: porcentaje

## DISCUSIÓN

Aunque se ha demostrado que las vacunas son eficaces en la prevención de enfermedades infecciosas y en el control de discapacidades, la indecisión ante las vacunas ha aumentado en todo el mundo en los últimos años, lo que ha provocado un descenso de las tasas de inmunización^(21)^. El SAGE subraya la importancia de proporcionar información a las personas implicadas en la prestación de asistencia sanitaria para prevenir la indecisión ante las vacunas y destaca la necesidad de estos esfuerzos informativos dirigidos a estos grupos^(4)^. En este contexto, los estudiantes de enfermería representan un grupo objetivo crítico para la educación sobre vacunas e inmunización, por lo que es esencial evaluar el impacto de las intervenciones educativas diseñadas para ese grupo demográfico^(3)^. Aunque varios estudios han evaluado el impacto de la educación sobre vacunas en estudiantes de enfermería de diferentes países^(11,22)^, la ausencia de este tipo de investigación en Turquía constituye una laguna en la bibliografía. Por lo tanto, este estudio tiene como objetivo evaluar el impacto de la educación sobre vacunas en los estudiantes de enfermería en Turquía en términos de sus actitudes hacia las vacunas y sus conocimientos relacionados con las vacunas.

En el estudio, se evaluó inicialmente el impacto de la educación sobre vacunas en las actitudes hacia las vacunas, y los resultados indicaron un aumento de las actitudes positivas de los estudiantes de enfermería hacia las vacunas tras la formación. El programa de educación sobre vacunas incluía temas como los descubrimientos de Edward Jenner y Jonas Salk, la reducción de los casos de enfermedades infecciosas y de las tasas de mortalidad tras el desarrollo de vacunas, imágenes de niños discapacitados por enfermedades infecciosas, los beneficios de las vacunas, las amenazas que supone el descenso de las tasas de inmunización para la salud pública y de los niños, y un caso reciente de rabia en el país en el que la no notificación de una mordedura de perro y la no vacunación provocaron la muerte. Se cree que estos temas incluidos en el programa de educación sobre vacunas han mejorado la actitud de los estudiantes de enfermería hacia las vacunas. Del mismo modo, la literatura contiene hallazgos que se alinean con nuestros resultados, informando de que los programas de educación sobre vacunas diseñados para estudiantes son eficaces para fomentar actitudes positivas con respecto a las vacunas^(7,22,23)^.

Varios modelos que pretenden explicar los procesos actitud-comportamiento sugieren que las actitudes son uno de los principales determinantes del comportamiento, y que fomentar actitudes positivas hacia un tema puede aumentar la probabilidad de comportamientos adecuados^(24)^. Las actitudes hacia las vacunas pueden influir no sólo en el comportamiento de vacunación de un individuo, sino también en las actitudes y comportamientos de los demás con respecto a la vacunación^(6)^. Las enfermeras y enfermeros con actitudes muy positivas hacia las vacunas pueden estar más dispuestas a asesorar a los padres y a las comunidades sobre las vacunas y a fomentar la vacunación. Al fomentar las actitudes positivas hacia las vacunas entre las enfermeras, más personas de la comunidad podrían recibir asesoramiento relacionado con las vacunas, lo que conllevaría beneficios significativos en la promoción de la vacunación y el aumento de las tasas de inmunización^(25)^. En este contexto, es crucial planificar iniciativas para mejorar la actitud de los estudiantes hacia las vacunas, concienciarlos sobre la gravedad de las enfermedades prevenibles mediante vacunación y proporcionar información sobre los beneficios de las vacunas.

Otro criterio de evaluación en el estudio fue el impacto de la educación sobre vacunas en los conocimientos relacionados con las vacunas. Los resultados del estudio revelaron que los conocimientos de los alumnos sobre las vacunas aumentaron significativamente tras el programa educativo. Durante las sesiones educativas sobre vacunas, se hizo especial hincapié en temas como los componentes de las vacunas, los mecanismos de acción, las enfermedades que previenen las vacunas, las normas generales de conservación de las vacunas, la preparación de los calendarios de vacunación infantil y los efectos adversos tras la inmunización. Se cree que los conocimientos detallados de los participantes en estas áreas contribuyeron a la mejora general de los conocimientos relacionados con las vacunas. De forma similar a nuestros hallazgos, estudios anteriores han informado de que los programas de educación sobre vacunas diseñados para estudiantes mejoran eficazmente los conocimientos relacionados con las vacunas^(11, 26, 27,28)^ . Tener conocimientos suficientes sobre las vacunas desempeña un papel fundamental a la hora de proporcionar información precisa durante las sesiones de educación y asesoramiento y de fomentar decisiones correctas sobre las vacunas. Garantizar que el personal de enfermería posea conocimientos adecuados sobre las vacunas es esencial para permitir que el público, en particular los padres, acceda a información precisa sobre las vacunas, abordar las dudas sobre las vacunas y mejorar las tasas de inmunización^(29)^. Además, dado que las enfermeras son a menudo los primeros profesionales sanitarios que se relacionan con la comunidad en los servicios de atención primaria^(10)^, es crucial planificar iniciativas destinadas a mejorar los conocimientos de los estudiantes de enfermería relacionados con las vacunas e incorporar la educación sobre vacunas en los planes de estudios de enfermería. Además, en consonancia con las recomendaciones del SAGE^(17)^, los programas de educación sobre vacunas también deberían abordar el creciente problema de las dudas sobre las vacunas y las amenazas para la salud pública que plantea^(17)^.

Además de las actitudes y los conocimientos sobre las vacunas, el estudio también examinó las percepciones de los estudiantes de enfermería sobre las vacunas y su confianza en ellas. Los resultados revelaron un aumento significativo de la proporción de participantes que confiaban en las vacunas y creían que las vacunas son una práctica beneficiosa tras la intervención educativa del estudio. El programa de educación sobre vacunas abarcó temas como los procesos de producción de vacunas, los estudios de fase, la supervisión posterior a la administración, la cadena de frío, el Sistema de Seguimiento de Vacunas implantado en Turquía y los beneficios que se consiguen con la vacunación. Se cree que la inclusión de estos temas en el programa influyó positivamente en la percepción que los estudiantes de enfermería tienen de las vacunas y en su confianza en ellas. Además, el aumento de los conocimientos de los estudiantes de enfermería relacionados con las vacunas y la mejora de sus actitudes hacia las vacunas durante el programa educativo pueden haber contribuido a mejorar sus percepciones y su confianza en las vacunas. Este hallazgo es especialmente significativo, ya que sugiere que la educación sobre vacunas puede reducir las dudas al respecto y reforzar la confianza en las vacunas. En la bibliografía se han descrito resultados similares, como que los programas de educación sobre vacunas reducen las dudas y aumentan la confianza de los estudiantes en las vacunas^(23,26)^. Además, los estudios han demostrado que las intervenciones educativas dirigidas a vacunas distintas de las inmunizaciones infantiles, como las vacunas contra el virus del papiloma humano (VPH) y COVID-19, mejoran la confianza de los estudiantes en las vacunas y reducen la indecisión en relación a las mismas ^(30,31)^. Teniendo en cuenta que los estudiantes de enfermería serán los futuros profesionales sanitarios y que unas enfermeras con altos conocimientos pueden contribuir a aumentar las tasas de inmunización, es esencial planificar iniciativas para mejorar su confianza en las vacunas. Además, la futura planificación de la educación sobre vacunas debería centrarse en aumentar la concienciación sobre los beneficios de las vacunas y los logros obtenidos con la vacunación, ya que esto desempeña un papel fundamental en el fomento de percepciones positivas de las vacunas y en el mantenimiento de la confianza en ellas. Esta intervención puede ser importante para animar a los padres y a la comunidad a vacunar a los estudiantes que serán responsables de prestar servicios de enfermería en el futuro, al aumentar su concienciación sobre la importancia de las vacunas.

## LIMITACIONES

Aunque los resultados del estudio contribuyen a la literatura, también presentan ciertas limitaciones. En primer lugar, el estudio se llevó a cabo con un grupo de estudiantes que se ofrecieron voluntariamente a participar en el programa de educación sobre vacunas, y no se realizó ninguna aleatorización. En segundo lugar, no hubo grupo de control en el estudio, lo que impidió comparar los resultados obtenidos del grupo experimental con un grupo de control. Además, la confianza en la vacuna se evaluó como una variable categórica. No se evaluó utilizando una herramienta de medición validada y fiable. La implementación del programa educativo en un formato tradicional complica la generalización de los resultados. Además, esta intervención se aplicó una sola vez y no se examinaron sus efectos a largo plazo. A pesar de estas limitaciones, los hallazgos son significativos, ya que demuestran que la educación sobre vacunas puede aumentar los conocimientos relacionados con las vacunas, mejorar las actitudes hacia las vacunas y contribuir a aumentar la confianza en las vacunas.

## CONCLUSIÓN

En este estudio, realizado para evaluar el impacto de la educación sobre vacunas en estudiantes de enfermería, se concluyó que los conocimientos relacionados con las vacunas aumentaron significativamente con un tamaño del efecto alto, y las actitudes hacia las vacunas mejoraron con un tamaño del efecto medio después de la intervención. Además, disminuyeron las dudas sobre las vacunas y aumentó notablemente la proporción de participantes que creían que las vacunas eran beneficiosas y necesarias. Con base en estos resultados, se rechazan las hipótesis del estudio (H0_1_: La educación sobre vacunas no tiene ningún efecto sobre las actitudes hacia las vacunas de los estudiantes de enfermería. H0_2_: La educación sobre vacunas no influye en los conocimientos sobre vacunas de los estudiantes de enfermería).

Teniendo en cuenta que los estudiantes de enfermería son futuros profesionales de la salud y que los enfermeros suelen ser la principal fuente de información para el público sobre vacunas y otros temas sanitarios, se recomienda planificar iniciativas educativas para mejorar sus conocimientos sobre vacunas. También se recomienda incorporar temas relacionados con las vacunas y la inmunización en los planes de estudios de enfermería. Además, sería crucial abordar el estado actual de la inmunización y las posibles amenazas para la salud pública que plantea el aumento de las dudas sobre las vacunas. Las investigaciones futuras podrían estudiar las dudas sobre las vacunas entre los estudiantes de enfermería y evaluar el impacto de la educación sobre las vacunas en ensayos controlados aleatorizados. Además, podrían evaluarse los efectos a largo plazo de la educación sobre vacunas en los conocimientos relacionados con las vacunas y las actitudes hacia las mismas.

## Data Availability

Los datos de la investigación están disponibles previa solicitud razonable al autor correspondiente.
